# Repositioning Brusatol as a Transmission Blocker of Malaria Parasites

**DOI:** 10.1021/acsinfecdis.4c00434

**Published:** 2024-10-01

**Authors:** Amelia Cox, Neelima Krishnankutty, Steven Shave, Virginia M. Howick, Manfred Auer, James J. La Clair, Nisha Philip

**Affiliations:** † School of Biodiversity, One Health and Veterinary Medicine, College of Medical, Veterinary and Life Sciences, University of Glasgow, Garscube Campus, Bearsden Road, Glasgow G61 1QH, United Kingdom; ‡ Institute of Immunology and Infection Research, University of Edinburgh, Ashworth Laboratories 2, Room 3.11, Edinburgh EH9 3FL, United Kingdom; § School of Biological Sciences, University of Edinburgh, The King’s Buildings, Edinburgh EH9 3BF, United Kingdom; ∥ Xenobe Research Institute, P.O. Box 3052, San Diego, California 92163, United States

**Keywords:** quassinoids, brusatol, malaria, transmission blocking, drug discovery, natural products

## Abstract

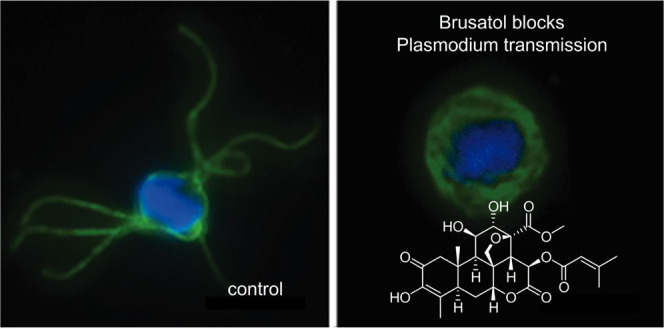

Currently, primaquine is the only malaria transmission-blocking drug recommended by the WHO. Recent efforts have highlighted the importance of discovering new agents that regulate malarial transmission, with particular interest in agents that can be administered in a single low dose, ideally with a discrete and *Plasmodium*-selective mechanism of action. Here, our team demonstrates an approach to identify malaria transmission-blocking agents through a combination of *in vitro* screening and *in vivo* analyses. Using a panel of natural products, our approach identified potent transmission blockers, as illustrated by the discovery of the transmission-blocking efficacy of brusatol. As a member of a large family of biologically active natural products, this discovery provides a critical next step toward developing methods to rapidly identify quassinoids and related agents with valuable pharmacological therapeutic properties.

The spread of drug resistance, including resistance to frontline therapies such as artemisinin, has become one of the leading concerns in treating malaria infections.^1,2^ While promising agents have been discovered that target the human liver (prophylatic) stages^3−6^ and blood (curative) stages,^7−9^ inhibiting transmission stages responsible for spreading parasites from humans to the mosquito vector is gaining increased interest.^10^ Unsurprisingly, to eliminate malaria globally, blocking parasite transmission is essential.^11^ Parasite transmission from human to mosquito is initiated by sexually dimorphic male and female gametocytes.^12^ Gametocytes circulating in human blood are taken up by the mosquito where the cells transform into male and female gametes in the mosquito midgut.^13^ Male gametes undergo three rounds of rapid mitotic genome replication within 10 min and concurrently polymerize tubulin to form axonemes. Eventually, the cell undergoes cytokinesis, and a single copy of the genome is packaged into flagellated gametes. Female gametes release translationally repressed mRNAs for protein expression.^14^ Fertilization of gametes results in the formation of a zygote, which develops into a motile, mosquito-infectious ookinete.^15^ The ookinete then migrates to the midgut wall to form an oocyst. The oocysts develop into thousands of sporozoites that travel to the salivary glands, poised to infect a new human host (Figure 1).

**Figure 1:**
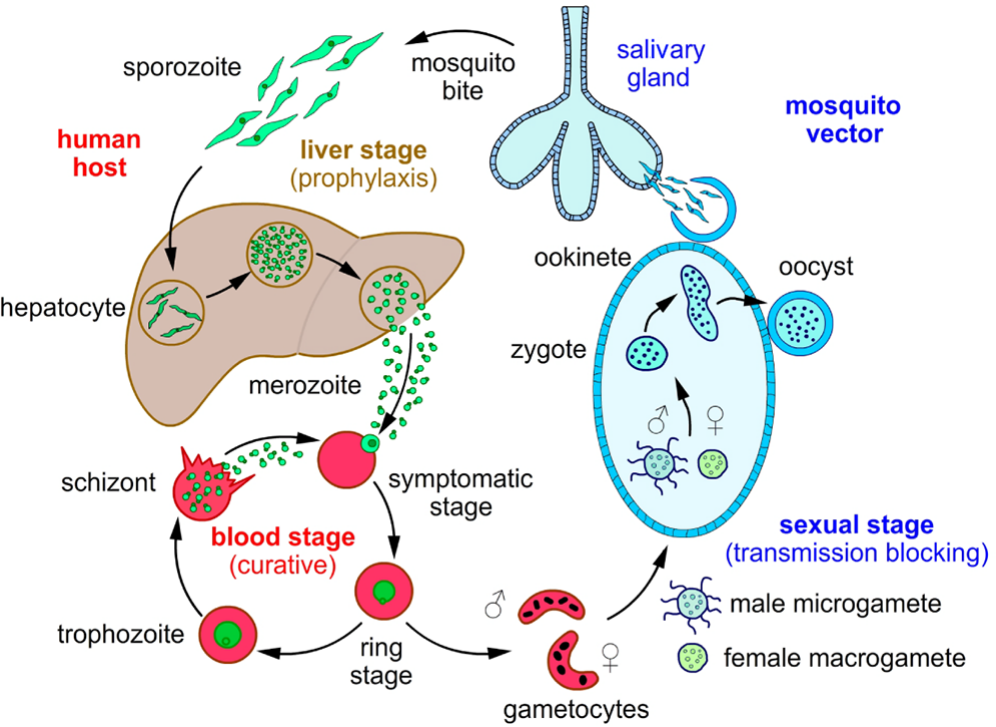
Malaria parasite lifecycle and potential stages for therapies. Infectious sporozoites enter the bloodstream from the saliva of a female anopheles’ mosquito and quickly invade liver cells. Liver stage development is a target for prophylaxis. After 7–10 days of differentiation and multiplication, merozoites are released into the bloodstream, where they invade circulating red blood cells. Over the next 48 h, parasites mature through ring, trophozoite and schizont stages; at this point, 8–32 new daughter cells are released to infect additional red blood cells. The pernicious stage is responsible for all symptoms attributed to malaria and is a target for curative therapies. Following this stage, either stochastically or in response to environmental conditions, few parasites exit the replicative cycle and follow a different developmental pathway and form sexually mature gametocytes. A mosquito taking a blood meal from an infected person ingests these gametocytes, and a complex sexual reproductive phase takes place in the mosquito midgut. After maturation and multiplication, sporozoites ultimately pass into the insect’s salivary glands and are injected into a new human host when the mosquito feeds. Gametocytes and early developmental stages in the mosquito are key targets for transmission blocking.

Transmission blocking strategies employing small molecules initially focused on killing gametocytes (gametocytocidal activity).^16,17^ However, recent studies have broadened the scope of blocking transmission by including targeting of early mosquito stage development.^18^ Such antimalarials can be administered to patients, where the compounds are delivered to the mosquito when it takes a blood meal. Currently, a priority for drug development is identifying dual-active compounds which can target both the sexual blood stages (curative) and the sexual stages (transmission blocking).^19−22^ To this end, we investigated whether a promising panel of natural products reported to have potent activity against asexual blood stage (ABS) parasites could also contain transmission-blocking capacity.

Our studies began by evaluating current approaches to screen for transmission blockers.^23−25^ Here, the goal of our study was to develop a screening platform that rapidly unites *in vitro* compound identification^26,27^ with *in vivo* evaluation.^28,29^ Early mosquito stage development assays in *P. falciparum* are challenging and few research groups have developed assays for efficient gametocyte activation.^30,31^ We were keen to exploit the rodent malaria model, *P. berghei*, where gametocyte activation and further ookinete development assays *in vitro* are reliable and highly efficient.^32^ To establish this workflow, we identified a training set of compounds that contained established and untested natural products with antimalarial activity. As shown in Figure 2, we selected five natural products that have reported antimalarial activity. This set included: a quassinoid, brusatol (**1**);^33^ an alkaloid, manzamine A (**2**);^34^ an isocyanoterpene, 7,20-diisocyanoadociane (**3**);^35,36^ a fungal metabolite hexacyclinol (**4**);^37,38^ and a diterpenoid, bromophycolide A (**6**).^39^ Here, we were interested in understanding if the reported antiplasmodial activity of these compounds also displayed transmission blocking potential. A second set of compounds without reported antiplasmodial activity was selected to represent the screening of unknown test fractions. Here, we included three compounds including: an antiproliferative macrocyclic peptide microsclerodermin D (**5**);^40,41^ a cytostatic fungal metabolite, laetirobin (**6**);^42,43^ and a cytokine-targeting spirotetronate, spirohexenolide A (**7**).^44,45^

**Figure 2:**
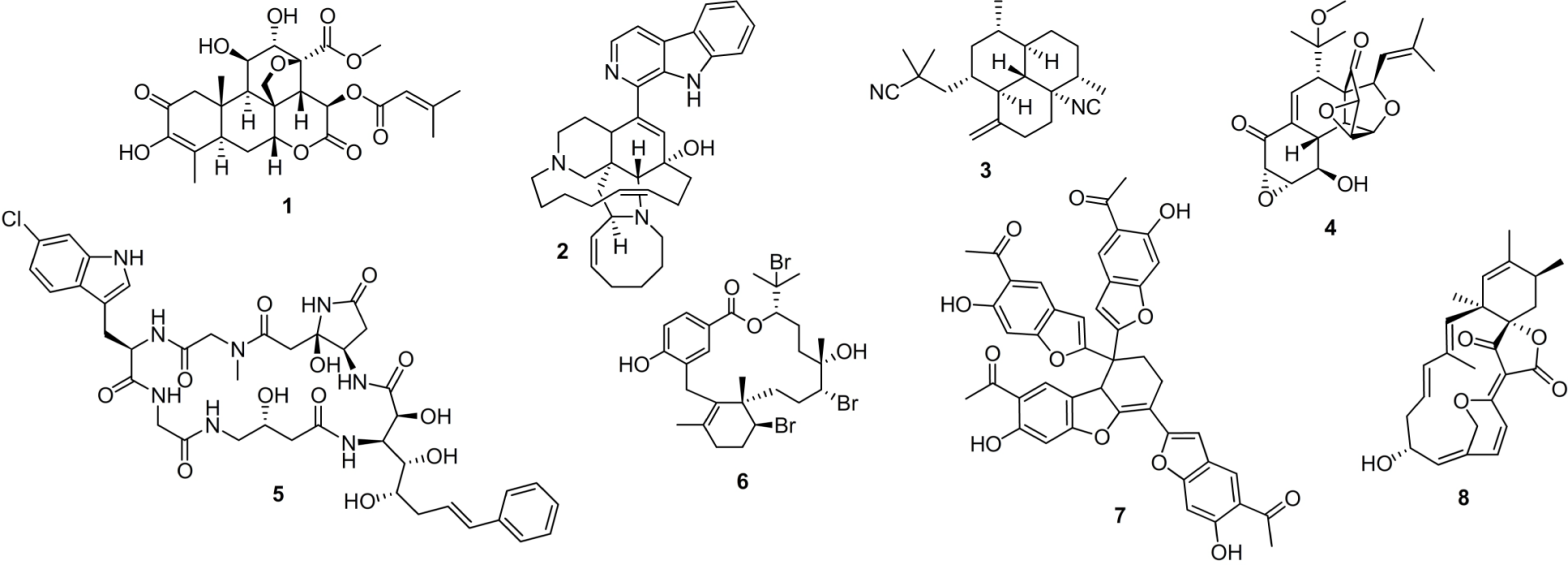
Structures of the five natural products (**1–4** and **6**) with demonstrated antiplasmodial activity and three (**5**, **7,** and **8**) with unexplored antiplasmodial activity.

Using the rodent malaria model, *P. berghei*, we assessed whether the compounds target mature gametocytes, sex-specific gamete function, or inhibited early stages in the mosquito. We identified brusatol (**1**) as a potent transmission blocker with as yet unreported multistage activity. Moreover, brusatol (**1**) was active *in vivo*, where a single dose can block transmission to the mosquito. Notably, we confirmed the transmission blocking activity of brusatol in the human malaria parasite *P. falciparum*. We believe that brusatol (**1**) shows viable activity as a transmission blocker and holds promise for further development as a dual-active antimalarial therapy.

## Results and Discussion

### Identification of Brusatol (**1**) and Manzamine A (**2**) as Inhibitors of *P. berghei* Transmission Stages

**Figure 3:**
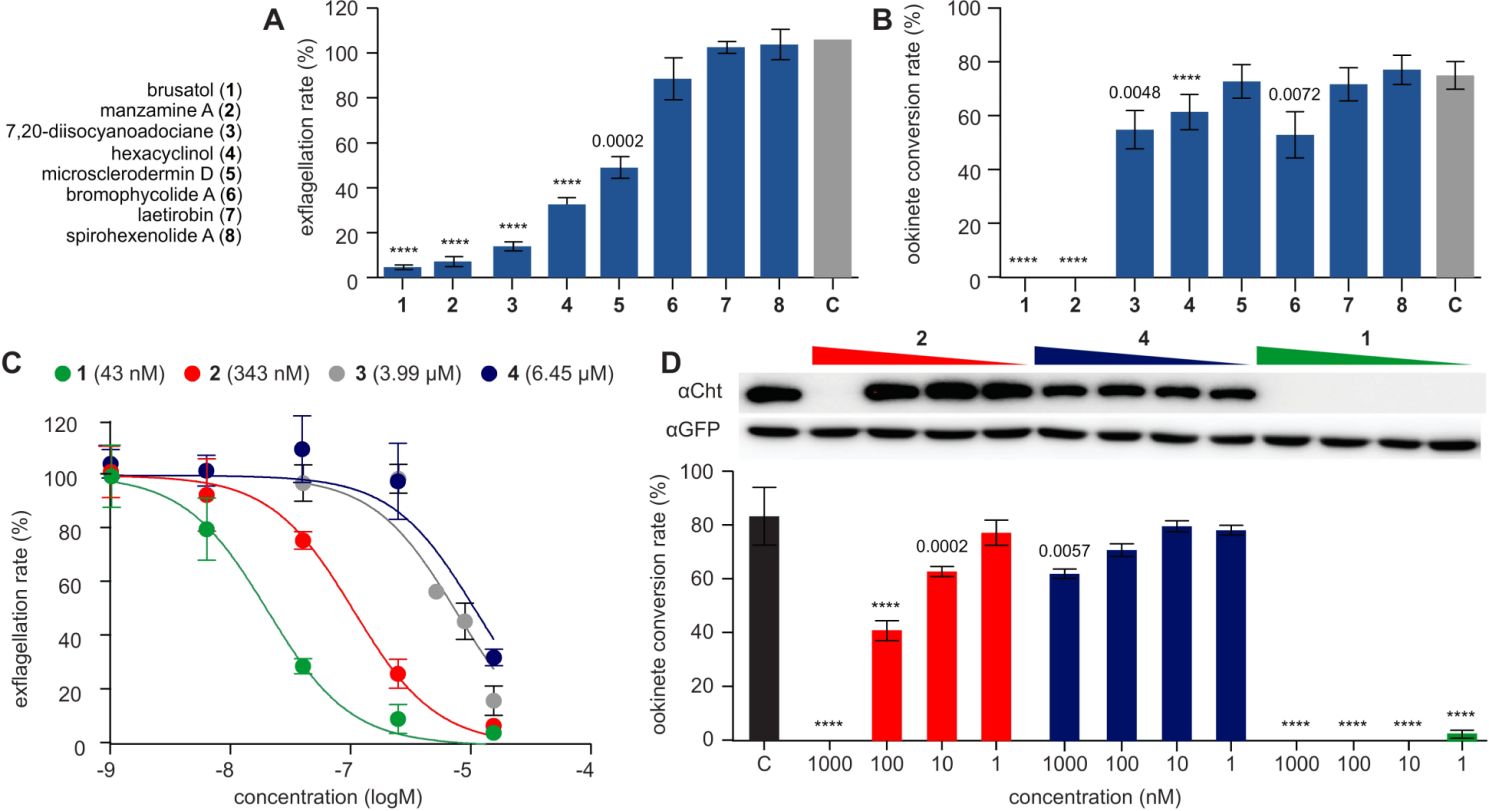
Transmission blocking capacity of selected natural products. Eight compounds were profiled to interrogate two key features of parasite transmission namely male gamete activity (exflagellation) and formation of the mosquito invasive form (ookinete). (A) Exflagellation rates observed in the presence of each compound at a concentration of 10 μM. (B) Ability to inhibit ookinete development by the eight compounds. (C) Dose response curves and IC_50_ values of compounds **1-4** showing potent exflagellation inhibition activity. (D) Dose response profiles of compounds of **1**, **2**, and **4** on ookinete development, which was assessed by expression of Chitinase protein, a feature of mature ookinetes (upper panel) and microscopy (lower panel). GFP was used as loading control. Standard deviations (error bars) and means are from four biological replicates. p-Values are given by **** < 0.0001 or numerically over each bar.

In a first step, we evaluated whether one of our natural product test sets inhibited male gamete activity or/and early mosquito stage development, both processes essential for successful parasite transmission. Mature *P. berghei* gametocytes were incubated with 10 μM of each compound for 30 min at 37 °C prior to initiating gamete formation. After 24 h of incubation in ookinete medium containing brusatol, we assessed the development of the mosquito-invasive ookinete. Four compounds showed potent activity against male gametes, including two that blocked ookinete development (Figure 3A–C). Notably, both manzamine A and brusatol inhibited male gamete exflagellation by more than 80% and completely blocked ookinete development. Although light microscopy is a powerful tool for evaluating developmental phenotypes, it is known that only a few ookinetes are sufficient to establish a mosquito infection. Consequently, we combined microscopy with sensitive Western blotting and probed for the abundantly expressed, ookinete-specific Chitinase. Although not essential for ookinete development, its chitin-degrading activity promotes parasite invasion of the mosquito midgut.^46,47^ Chitinase protein expression commences between 14 and 16 h post-gametocyte activation, and in hexacyclinol (**4**), manzamine A (**2**), and brusatol (**1**) treated cells, Chitinase expression was undetectable (Figure S1A,B). Although Chitinase expression is not dose-responsive, it is a sensitive marker for detecting post-fertilization development (Figure 3D). Curiously, hexacyclinol activity suggests the compound might directly impact Chitinase protein expression without analogous decrease in ookinete conversion. Importantly, brusatol (**1**) showed the highest potency against male gametes (IC_50_ value of 43.0 ± 1.1 nM) and onward ookinete development (Figure S1C,D).

### Phenotypic Validation of Brusatol as a Multistage Transmission Blocker in *P. berghei*

**Figure 4:**
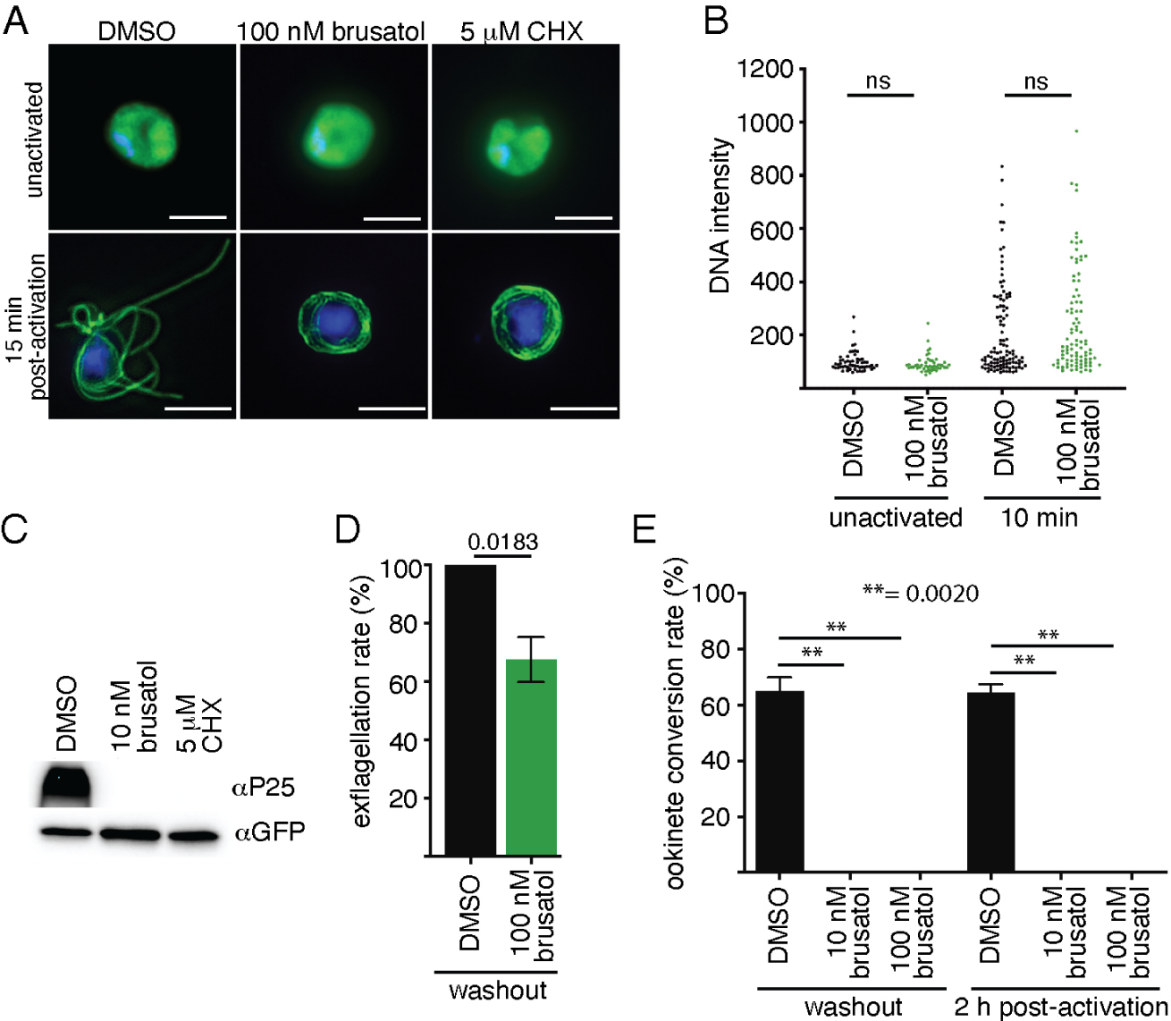
Brusatol exhibits multistage activity in *P. berghei*. Gametocytes were treated with 100 nM for 30 min and gamete production was initiated by a drop in temperature and addition of ookinete media containing brusatol. (A) DMSO and brusatol-treated unactivated male gametocytes display similar nuclear (DAPI, blue) and homogeneous α-tubulin staining (green). Fifteen minutes post-activation, DMSO-treated males polymerize tubulin to form axonemes and release up to eight flagellated gametes. Brusatol-treated gametocytes can polymerize tubulin, but are unable to produce viable gametes, (**B**) Genome replication is unaffected in brusatol-treated gametes. (**C**) Brusatol-treated female gametes cannot initiate translation of stored mRNA transcripts such as P25 in *P. berghei*. Cycloheximide (CHX), a protein translation inhibitor shows similar activity to brusatol. (**D**) Brusatol action on male gametes is partially reversible when compound was absent in ookinete media (wash-out) (**E**) Both wash-out and exposure to brusatol 2 h post-activation severely compromises ookinete development.

To investigate the potential mechanism of action of brusatol, we performed molecular and cellular phenotyping assays on gamete and zygote function. Gametocytes were treated with 100 nM of brusatol for 30 min at 37 °C, and the compound was carried over in ookinete medium. Brusatol and DMSO-treated male gametocytes displayed similar nuclear morphology, tubulin distribution and DNA content (Figure 4A,B). Activated males underwent genomic replication and formed microtubular axonemes but failed to exflagellate and form mature gametes (Figure 4A,B).

The higher potency of brusatol on ookinete development in comparison to male gamete function raises the intriguing prospect of multistage activity. Inhibition of ookinete formation could result from defective male or female gametes or from obstruction of zygote development following gamete fertilization. We first investigated whether brusatol showed activity against female gametes. Temporally regulated translation of stored mRNA transcripts is a hallmark of female gamete and ookinete development. One of the first transcripts released for protein translation is P25, a protein trafficked to the parasite surface and promotes ookinete survival in the mosquito midgut epithelium.^48,49^ Gametocytes were treated with 10 nM brusatol, and 2 h post-induction with brusatol-containing ookinete media, cells were harvested and examined for P25 expression. Similar to treatment with the potent protein synthesis inhibitor, cycloheximide (5 μM), brusatol completely blocked P25 protein expression (Figure 4C).

Inhibition of gamete function by brusatol could be a consequence of killing or damaging gametocytes and/or directly preventing gamete activation.^50^ Following a 30-min pulse of brusatol or DMSO, gametocytes were activated with ookinete medium in the absence of the compound. While male gamete activity was partially restored, no mature ookinetes were recovered revealing differential susceptibility between males and females (Figure 4D,E). Moreover, the potential of brusatol to inhibit protein synthesis (Figure 4C), suggests zygote (post-fertilization) development could be vulnerable. Indeed, when brusatol was added 2 h post-activation, no mature ookinetes were recovered (Figures 4D and S2).

Further investigation is required to precisely determine the mechanism of the brusatol activity. However, previous studies in mammalian cells^51^ and our observations here suggest that brusatol acts in *Plasmodium* by inhibiting protein synthesis.

### Confirmation of Transmission Blocking Activity in *P. falciparum*

**Figure 5:**
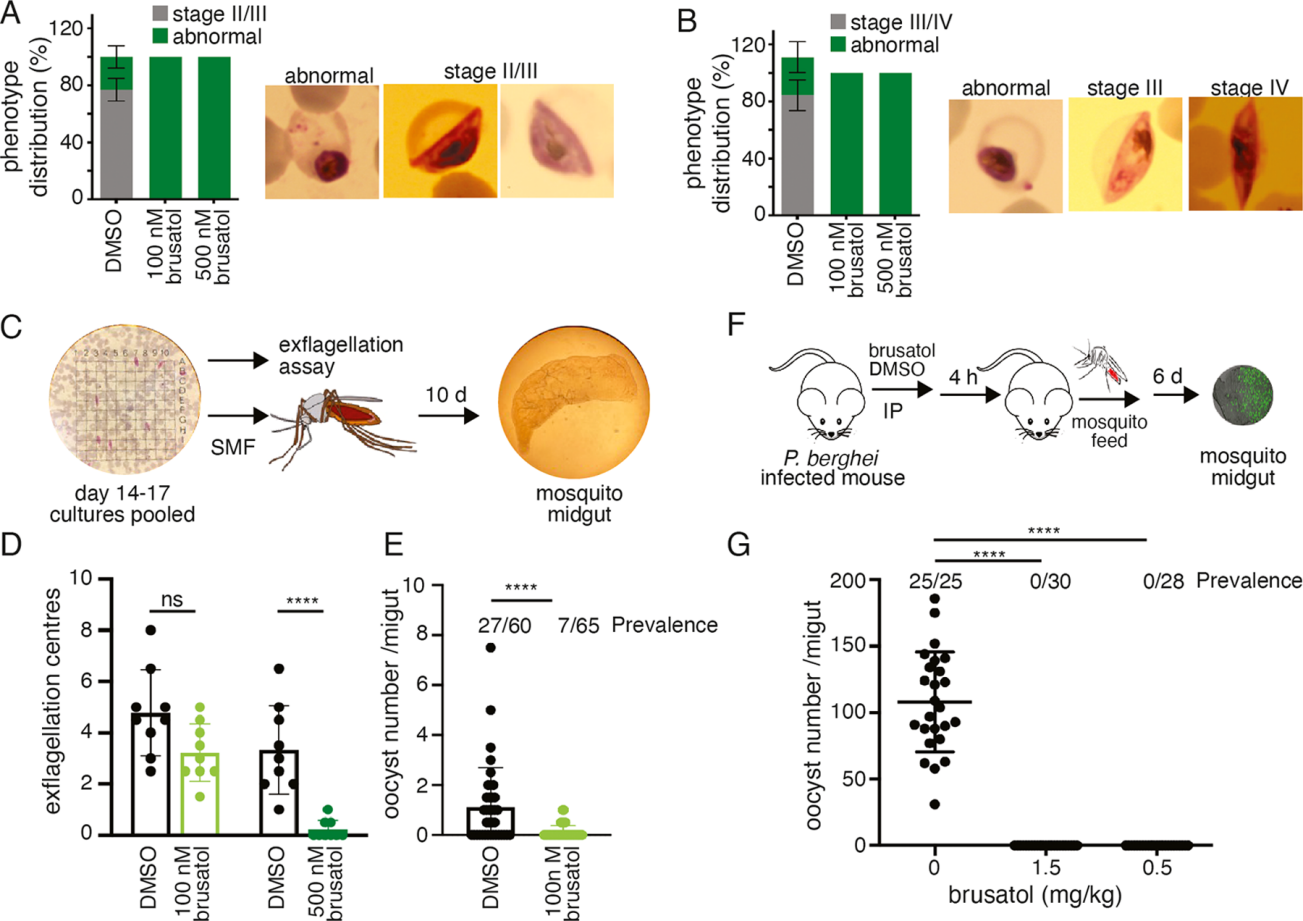
Transmission blocking effect of brusatol. Brusatol exhibits potent gametocytocidal activity on multiple developmental stages in *P. falciparum*. (A) Incubation of stage I gametocytes with brusatol inhibited further maturation to stage II/III (*n* = 3). (B) Brusatol exposure in stage II/III prevented transition to stage III/IV (*n* = 3). (C) Transmission blocking activity of brusatol in *P. falciparum* was assessed by first incubating pooled day 14–17 gametocytes for 1 h with the indicated brusatol concentrations, exflagellation rates measured and subsequently a standard membrane feed (SMF) assay was used to determine the ability to establish mosquito infections. (D) Exflagellation rates were significantly reduced with 500 nM exposure. Variation in the two DMSO treated exflagellation assays are likely a result of different serum batches. (**E**) oocyst numbers and prevalence in the mosquito were diminished even at the lower concentration of 100 nM suggesting a multistage mode of action. (**F** and **G**) *In vivo* transmission blocking activity of brusatol. Mice were infected with 10^5^ parasites. On day 3 of infection, mice were administered a single dose of brusatol at 1.5 mg/kg, 0.5 mg/kg, or DMSO vehicle control by IP injection. Four hours post-IP, *Anopheles stephensi* were allowed to feed on infected mice. Six days post-feed, mosquitoes were dissected (at least 25 per condition) and oocyst numbers and prevalence determined by microscopy. ****p < 0.0001.

To establish whether brusatol exposure results in the inhibition of human malaria parasites, we first assessed the activity of brusatol on gametocyte development. In contrast to 24–32 h development in *P. berghei*, gametocyte maturation of *P. falciparum* takes ∼12 days and transitions through distinct morphological and molecular phases (I–V).^52^ To determine the activity of brusatol on gametocyte maturation, we exposed stage I and stage II/III gametocytes to the compound (100 nM or 500 nM) and, 24–48 h post-incubation, examined the transition to the next developmental stage. Complete inhibition of gametocyte development was observed, and neither stage I nor II/III stages progressed to the subsequent maturation phase (Figure 5A,B), revealing strong gametocytocidal activity.

Notably, brusatol demonstrated potent activity against mature gametocytes in *P. berghei* and to uncover if the compound similarly affected human malaria parasites, we examined infectivity of treated *P. falciparum* (3D7) gametocytes in the mosquito *Anopheles gambiae* (Kisumu) (Figure 5C). Gametocyte cultures, matured to stage V over a period of 14–17 days, were counted for gametocytemia and then incubated with brusatol (100 or 500 nM) or DMSO for 1 h at 37 °C. Gametocytes were activated, and their ability to exflagellate was measured by counting exflagellation centers. Marked reduction in the number of exflagellating centers was observed upon brusatol treatment and a concentration of 500 nM resulted in >80% inhibition of male gamete activity (Figure 5D). Although effective at higher concentrations, brusatol activity in *P. falciparum* replicates the phenotype seen in *P. berghei* and suggests that compound exposure impairs male fertility.

Inhibition of *P. falciparum* male gamete activity upon brusatol exposure suggests that the compound will block parasite transmission to the mosquito. Brusatol (100 nM) or DMSO exposed gametocytes were fed to mosquitoes, and 235 mosquito midguts were viewed at 10 days post infectious feed for presence of oocysts (Figure 5E). A significant reduction in oocyst burden (*p* ≤ 0.0001) and infection prevalence was observed with brusatol treated gametocytes (*p* ≤ 0.0001). Taken together this data demonstrates the ability of brusatol to block transmission is conserved across parasite species.

### *In Vivo* Validation of the Transmission Blocking Activity of Brusatol

Following the potent activity of brusatol on *P. berghei* and *P. falciparum* gametes *in vitro*, and the subsequent block of *P. falciparum*transmission to mosquitoes, we assessed if brusatol demonstrated activity *in vivo* (Figure 5F). To validate the transmission blocking activity of brusatol *in vivo*. *P. berghei*infected mice were administered a single intraperitoneal (IP) dose of 1.5 and 0.5 mg/kg of brusatol or DMSO, 4 h prior to the mosquito feed. At day 6 post-feed, transmission blocking activity was determined by assessing both oocyst prevalence and intensity. Control feeds averaged 95–100% oocyst prevalence averaging 100 oocysts/midgut. Remarkably, while brusatol exposure had no effect on the mosquito survival rate, a low 0.5 mg/kg dose resulted in a complete block of parasite transmission (Figure 5G; S3).

Brusatol, a plant (*Brucea javanica*)-derived triterpene lactone, belongs to a large family of natural products isolated from Simaroubaceae^53^ that include the bruceanols,^54,55^ bruceolides,^56^ eurycomanone,^57^ gutolactones,^58^ isobruceins,^59^ neoquassin,^60^ quassimarins,^61^ samaderines,^59^ and simalikalactones.^62,63^ Extracts of *Brucea javanica* are a well-known Chinese herbal medicine, traditionally used in Chinese medicine for the treatment of intestinal inflammation, diarrhea, malaria and cancer.^51^ Developing on the early work of Steele and colleagues in London,^64^ many of these quassinoids have shown antimalarial activity.^65−67^ Although some data exist from preliminary SAR studies,^68−70^ a detailed understanding of the mode and mechanisms of their transmission blocking activity have yet to be identified. In addition to antimalarial activity, brusatol has been explored for its anticancer activity.^71^

Initially explored for its antileukemic properties,^72^ its ability to inhibit NRF2,^73^ a key transcription factor regulating oxidative homeostasis, by enhancing protein ubiquitination leads to a disrupted redox balance. This ultimately results in a tumor cell death and tumor suppression. While other mechanisms have been suggested for this activity,^71^ these along with systemic toxicity and complex side effects observed during early phase clinical trials (nausea and vomiting) complicated clinical translation.

While brusatol is commercially available, the availability of derivatives is limited to a handful of natural products. Substructure searching of commercial databases reveals seven derivatives available at the time of writing, with bruceantin, bruceine A, and bruceantinol being the most widely offered, followed by bruceine B and D, and finally yadanzioside I and F. Attempts at scaffold hopping and identification of similar compounds through cheminformatics methods were unsuccessful, with no molecules prioritized for follow up purchase and assay, likely due to the high complexity of this natural product. Additionally, cheminformatic fragmentation using the breaking of retrosynthetically interesting chemical substructures (BRICS)^74^ and Retrosynthetic Combinatorial Analysis Procedure (RECAP)^75^ method yield no useful fragments for scaffold hopping, derivatization, or warhead optimization, returning only the core brusatol molecule, 3-methylbut-2-enal, methanol, propane, formaldehyde, and acetaldehyde (see Supporting Information). Not surprisingly, this is indicative of the unique structural features within brusatol.

## Conclusions

Overall, this study developed a straightforward pipeline for the discovery of plasmodial transmission blockers using a combination of the rodent malaria model *P. berghei* and the clinically relevant *P. falciparum*. Initially, *ex vivo* assays employing *P. berghei* allowed efficient compound evaluation in a systematic manner where multiple stages of parasite development were rapidly assayed, permitting several transmission blocking opportunities to be profiled. This allowed prioritizing compounds to be taken forward for testing in *P. falciparum*. Culturing of human malaria parasites requires containment level-3 (CL-3, with derogation) facilities in the UK and mosquito transmission studies in several European countries require CL-3 establishments. Moreover, production of viable gametocytes for transmission blocking assays is inefficient and lengthy (14–17 days vs 5 days in *Pb*). Using our efficient pipeline, we selected brusatol, which exhibited a strong gamete targeted activity at low nanomolar concentrations. Not only was brusatol’s activity on *P. berghei* male gametes validated on *P. falciparum* gametocytes where brusatol exhibited potent activity against male gamete function, and subsequently inhibited transmission to the mosquito, we also uncovered that brusatol prevents *P. falciparum* gametocyte maturation. Importantly, brusatol shows potent *in vivo* efficacy, where a single dose at 0.5 mg/kg completely blocked onward parasite transmission in the mosquito.

The strong activity of brusatol on post-fertilization development of ookinetes uncovers the potential of delivering the compound directly to mosquitoes through toxic sugar baits or on bed nets.^76^ To further progress brusatol derivatives toward clinical development, it will be necessary to overcome systemic toxicity and complex side effects. With a single dose LD_50_ value of 16.2 mg/kg in mice,^77^ human applications of brusatol remain very limited. Although this study identified activity at nM levels, future efforts will have to focus on understanding the critical structure activity relationships (SARs) associated with transmission blocking activity within the quassinoid family.^58,62,65,78^ Here, one can apply already known synthetic methods to evaluate analogues of brusatol,^33,79,80^ or other available quassinoids such as quassin.^81−83^ The goal of this effort would rely on the discovery of the molecular mechanism in which brusatol and associated analogues block plasmodial transmission. With this knowledge at hand, one can begin to adapt medicinal chemical methods for on-target optimization and reduced toxicity.^84^

While we did not anticipate the discovery of a viable hit within our initial training set, the focus of this study was to develop and validate an effective process based on the *in vitro* and *in vivo* assays to screen for transmission blockers exemplified in this work. Efforts are now underway to expand this assay to a high-throughput format for screening natural and synthetic test fractions with particular interest being placed on evaluating extracts from diverse species of *Brucea*.

## Methods

### Compounds

All materials were purified to >98% purity by preparative thin-layer chromatography (p-TLC) and checked by ^1^H- and ^13^C NMR prior to use. These materials were selected from a natural product library based on their known activity against plasmodia including brusatol (**1**), manzamine A (**2**), 7,20-diisocyanoadociane (**3**), hexacyclinol (**4**), and bromophycolide A (**5**). This set was supplemented with three compounds with unscreened plasmodial activity including: microsclerodermin D (**5**), laetirobin (**6**), and spirohexenolide A (**7**). All compound stocks were prepared in DMSO at 10 μM and stored at −20 °C until used.

### Plasmodia Strains

*P. berghei* experiments performed with the 507cl1 line, which constitutively expresses GFP under the eef1α promoter.^85^*P. falciparum* 3D7 strain was used for *Pf* gamete activation and standard membrane feed assays (SMFA).

### *P. berghei* Animal Infections

All animal work was performed in accordance with the UK Animals (Scientific Procedures) Act 1986 (amended in 2012) and the European Directive 2010/63/EU on the Protection of Animals Used for Scientific Purposes. All procedures were approved by the University of Edinburgh’s animal welfare and ethics board and UK’s Home Office (project license: PPL P04ABDCAA6). *P. berghei* parasites were maintained in CD-1 mice (Charles River) weighing between 22 and 30 g. Parasite infections were established by intraperitoneal injection (IP) of 200 μL of cryopreserved 507cl1 parasite stock. Mice were pretreated with 100 μL of phenylhydrazine at 12.5 mg/mL in physiological saline 48 h before infection to induce reticulocytosis. Asexual blood stage (ABS) parasites were cleared by administering sulfadiazine (25 mg/L) in drinking water. Infected red blood cells were collected by a cardiac puncture under terminal anesthesia.

### *P. berghei* Gamete Formation and Ookinete Development Assay

Infected red blood cells with a minimum 2% gametocytes were incubated with the reported concentration of each compound in Suspension animation or SA medium (RPMI with 25 mM HEPES, 5% FBS, 4 mM sodium bicarbonate, pH 7.2) for 30 min at 37 °C in a 50 μL volume. Male gamete activity was assayed by adding 500 μL of activation media/ookinete (RPMI with 25 mM HEPES, 20% FBS 4 mM sodium bicarbonate, and 100 μM xanthurenic acid, pH 7.8) at 21 °C. Twenty minutes post-activation, a 10 μL aliquot was used to count exflagellation centers on a hemocytometer with a 10× objective and percentage inhibition of each compound was determined by comparison to DMSO-treated cells. Two replicates of nine 0.1 mm^3^ fields with 20–30 cells/nL were counted for each biological repeat. The remaining activated cells were allowed to develop in the dark for 24 h in flat-bottom plates to form mosquito infective ookinetes. Ookinete conversion was assessed by Giemsa staining using light microscopy and conversion rates were determined by comparing mature ookinete (MO) numbers to total number of round (RO), retort (RE) and mature ookinetes (. A total of 300 cells across multiple fields were counted. The proportion of arrested or defective cells was calculated employing the same equation and replacing the numerator to the corresponding cell type. For washout experiments, gametocytes were incubated for 30 min with the indicated concentrations of brustaol or DMSO, washed twice in SA media, and activated with ookinete media. The ookinete conversion rate was calculated as previously described. To determine the activity of brusatol on fertilized cells, 50 μL IRBCs containing gametocytes were triggered with 1 mL of ookinete media and 2 h post-activation, 1 mL of ookinete media containing 20 nM, 200 nM, or DMSO was added to fertilized zygotes. Ookinete conversion was assessed as previously described

### *P. berghei* Genome Replication Assay in Male Gametes

Gametocytes were enriched from IRBCs using 55% nycodenz wt/vol in SA medium. 50 μL of purified gametocytes was incubated with 100 nM brusatol or DMSO for 30 min in SA at 37 °C. Cells were subsequently activated at 21 °C with the addition of 250 μL of ookinete media supplemented with 100 nM brusatol or DMSO. Ten min post-activation, cells were fixed in 4% PFA/PBS for 15 min, smeared on glass slides, dried, rinsed once in PBS and, mounted using Fluoromount-G with DAPI (00–4959–52, ThermoFisher Scientific). Images were captured on a Zeiss Axio Imager Z2 at 63× magnification, 25 stacks with step sizes of 0.2 μM. Stacks were projected by summing the slices and DNA intensity was calculated using Cell Profiler.^86^ DNA intensity was calculated for a minimum of 48 gametocytes at 0 min (unactivated) and 90 gametes at 10 min (activated).

### *P. bergheiIn Vivo* Transmission Blocking Assay

Six- to nine-week-old CD-1 mice were infected with *P. berghei* parasites by IP and infection was monitored by thin blood smears to quantify both parasitemia and gametocytemia. Mice were divided into groups of two and administered brusatol (0.5 and 1.5 mg/kg) or vehicle control (10% DMSO in phosphate buffer saline, pH 7.4). Four hours later, *Anopheles stephensi* mosquitoes were allowed to feed on brusatol-treated mice for 10 min. Mosquitoes were maintained on fructose/PABA for 6 days. Midguts were dissected, imaged on a Leica M205FA fluorescence stereomicroscope and oocysts counted by semiautomated analysis using ImageJ. Mosquito survival assays were also performed during the transmission blocking assay where survival rate was monitored every 3 days until 33 days post exposure. Data were analyzed on GraphPad prism using the Kaplan–Meier test.

### *P. falciparum* Gametocyte Development Assay

Parasites (*P. falciparum* NF54/3D7) were cultured in complete media (RPMI with 25 mM HEPES, 100 mM hypoxanthine, 24 mM sodium bicarbonate, 0.5% Albumax II, 5% human serum, and 25 μg/mL gentamicin), and rings were synchronized with 5% D-sorbitol. Induction was performed in early trophozoite stage (1.5% parasitemia, 5% hematocrit) by substituting the medium with minimal fatty acid, MFA medium (3.9 mg/mL fatty acid-free BSA, 30 μM palmitic acid, and 30 μM oleic acid in RPMI medium supplemented with 25 mM HEPES, 50 mg/L hypoxanthine, 2 mM glutamine, and 25 μg/mL gentamicin). After 24 h, medium was substituted with complete RPMI with 10% serum (excluding AlbumaxII). Subsequently, 24 h later, cultures were treated with N-acetyl glucosamine (NAG) to eliminate asexual stages. One day after NAG addition, cultures were examined for the presence of stage I gametocytes using Giemsa stain. Either stage I or stage II/III gametocyte cultures consisting of 2 mL volume were transferred to 6-well plates. Parasites were exposed to concentrations of 100 nM or 500 nM brusatol or DMSO control. After 48 h, gametocyte morphology and numbers were examined by Giemsa smears.

### *P. falciparum* Gamete Activation and Standard Membrane Feed Assay

Gametocytes were seeded from asexual *P. falciparum* 3D7 previously described.^87^ Briefly, an initial concentration of between 0.5% and 0.7% parasitized human red blood cell was cultured with 6% hematocrit in complete RPMI (RPMI medium with 25 mM HEPES 50 mg/L hypoxanthine, 2 g/L sodium bicarbonate, and 10% human serum) at a total volume of 5 mL. Red blood cells were obtained from the Scottish National Transfusion Bank and serum from Interstate Blood Bank (USA) or Pan BioTech UK. When parasitemia of >4%, was reached, the culture volume of complete RPMI was increased to 7.5 mL. Replicate cultures were set up 3 days after the induction of the first flasks of parasites. Mature gametocytes from 14- and 17-days post-induction were pooled, centrifuged, and incubated with 100 nM or 500 nM brusatol, or control DMSO, for 1 h at 37 °C. Subsequently, RPMI was removed and blood meals of 1 mL with 0.7% gametocytemia was prepared using erythrocytes and human serum (40:60 ratio) with the indicated brusatol concentrations. The same blood meals were used for assessing both gamete activity and mosquito transmission assays. To calculate male gamete activity, 15 μL sample of each bloodmeal was added to 225 μL of ookinete media with brusatol at 100 nM, 500 nM, or 0.003% DMSO at 22 °C, and 15 min later exflagellation was viewed on a hemocytometer. The number of exflagellating centers was recorded, and comparisons were made between brusatol-treated and paired control (DMSO) parasites.^88^ Statistical significance was determined using Poisson regression generalized linear models (glm) in R version 4.2.3, GUI 1.79, Big Sur ARM build (8198). The packages used included R base packages and effects_4.2–2. GLMs were employed to compare exflagellation rates between each brusatol concentration and the paired DMSO negative control.^89^ The models included treatment and culture as factors, with the link function specified as log.

Exflagellation inhibition assay (EIA) percentages were calculated using the following equation: E_I_ = (E_C_ – E_T_/E_C_) × 100, where E_c_ and E_T_ are the number of exflagellation centers in the control (DMSO-treated) and in the brusatol-treated samples, respectively, and E_I_ is exflagellation inhibition presented as a percentage reduction compared to the control.^89^ For mosquito transmission experiments, bloodmeals of the prepared volume, as described previously, were fed to 12-h-starved, virgin *A. gambiae* (Kisumu) female mosquitoes aged 4–7 days, using a standard membrane feeding assay.^90^ Mosquitoes were allowed uninterrupted blood access through the membrane for 30 min before removal. Following infected blood feed, any nonfed females were removed. The remaining fed females were housed at 26–28 °C and 70–80% humidity with continuous access to 5% glucose in PABA for 10 days. After 10 days, mosquitoes were killed by 70% ethanol exposure and immediately placed in DPBS on ice. Midguts were dissected out and viewed under 10× and 40× magnification for the presence of oocysts. The number of oocysts was recorded for each mosquito midgut, and the prevalence and intensity of infection were calculated. Statistical tests were performed in R (4.2.3 GUI 1.79 Big Sur ARM build (8198). Packages used included R base packages, effects_4.2–2,^91^ ggplot2_3.4.3.^92^ Binomial generalized linear models were used to compare each brusatol concentration to the paired DMSO-treated oocysts to determine if they were statistically significant. The number of infected mosquitoes was analyzed, with treatment and culture included as factors in these models.

### Immunofluorescence and Western Blotting

Gametocytes were activated as previously described. Twelve min post-activation, they were fixed with 4% paraformaldehyde in PBS and transferred to glass slides. The fixed cells were washed 3 × 10 min with PBS, permeabilized with 0.1% Triton X-100 PBS solution for 10 min, blocked in 3% BSA PBS solution for 1 h at room temperature, and incubated with α-tubulin antibody,^93^ at 1:3000 dilution in 3% BSA overnight at 4 °C. Cells were incubated with Alexa-488 labeled antimouse IgG secondary antibody (A-11029, ThermoFisher Scientific) and mounted with Fluoromount-G with DAPI (00-4959-52, ThermoFisher Scientific). Images were captured on a Zeiss Axio Imager Z2 and analyzed by ImageJ. For Western blotting to assess P25 expression, sulfadiazine-enriched gametocytes were treated with the indicated compounds as described in the gamete induction and ookinete development assay, except that the cultures were scaled up to 3 mL with a proportional increase in IRBCs. Proteins were extracted at 2 h (for assessing inhibition of protein synthesis) or 24 h post-activation (for ookinete development assay) in RIPA lysis buffer (50 mM Tris-HCl, pH 7.5, 150 mM NaCl, 2 mM EDTA, 1% NP-40, 0.1% SDS) supplemented with protease inhibitor cocktail (Roche). The lysate was iced for 20 min and centrifuged at 14000*g* for 15 min at 4 °C. The supernatant was heated in SDS sample buffer at 70 °C for 15 min, separated on a 4–15% gradient Tris-HCl gel, and transferred to a nitrocellulose membrane. The membrane was blocked with 5% skim milk in PBST buffer for 1 h at room temperature and then incubated with primary antibodies overnight at 4 °C. Antibodies used were α-Chitinase^94^ at 1:4000, α-P25 (peptide: CEDGF KLSIE ENK, rabbit polyclonal, Proteintech) at 1:4000 and α-GFP (monoclonal 13.1 + 7.1, Roche) at 1:3000 dilution. The membrane was washed three times for 15 min each with PBST and then incubated with HRP-conjugated secondary antibodies (antimouse IgG, 7076S and antirabbit IgG, 7074S from CST) at a 1:5000 dilution for 1 h at room temperature. The membranes were washed three times with PBST followed by a final wash with PBS, and proteins were visualized using an ECL advanced kit (GE healthcare).

### Cheminformatics Analysis and Fragmentation

Python (v 3.7.13) was used, along with RDKit (version 2022.3.2), to apply the BRICS and RECAP fragmentation methods. An RDKit Mol was generated using the SMILES string (below) for brusatol, which was passed to the RDKit-included implementation of BRICS and RECAP. Fragments were output in both 2D and SMILES representations. In addition, a helper function queried the CADD Group Chemoinformatics Tools and User Services to retrieve IUPAC names.

The Python code used to carry out fragmentations is available in a GitHub gist at:

https://gist.github.com/stevenshave/934c3c9f13d4affcae3e9d41c4cb19f8.

Smiles string for brusatol (**1**):

CC1 = C(C(=O)C[C@]2([C@H]1C[C@@H]3[C@]45[C@@H]2[C@H]([C@@H]([C@]([C@@H]4[C@H](C(=O)O3)OC(=O)C=C(C)C)(OC5)C(=O)OC)O)O)C)O

## Supporting Information Available

General experimental procedures; protocols for the *in vitro* and *in vivo* transmission assays; and cheminformatics evaluation of brusatol (PDF)
