# Supplementary material for: Repositioning Brusatol as a Transmission Blocker of Malaria Parasites
Source: ACS Infect Dis. 2024 Oct 1;10(10):3586–96. doi: 10.1021/acsinfecdis.4c00434 (PMC11474950; doi:10.1021/acsinfecdis.4c00434)
Supplement: Supplementary file 1 — id4c00434_si_001.pdf [file id4c00434_si_001.pdf]

SUPPORTING INFORMATION

Repositioning Brusatol as a Transmission Blocker of the Malaria Parasite

Amelia Cox, [1] Neelima Krishnankutty,[2] Steven Shave, [3] Virginia M. Howick, [1] Manfred Auer,[3,4] James J. La Clair[4,*] and Nisha Philip,[2,*]

[1] School of Biodiversity, One Health and Veterinary Medicine, University of Glasgow, College of Medical, Veterinary and Life Sciences, Garscube Campus, Bearsden Road G61 1QH United Kingdom

[2] Institute of Immunology and Infection Research, University of Edinburgh, Ashworth Laboratories 2, EH9 3FL, United Kingdom

[3] School of Biological Sciences University of Edinburgh, The King's Buildings, Edinburgh EH9 3BF, United Kingdom

[4] Xenobe Research Institute, P. O. Box 3052, San Diego, California, 92163, United States

Table of Contents

Figure S1 S2
Figure S2 S3
Figure S3 S4

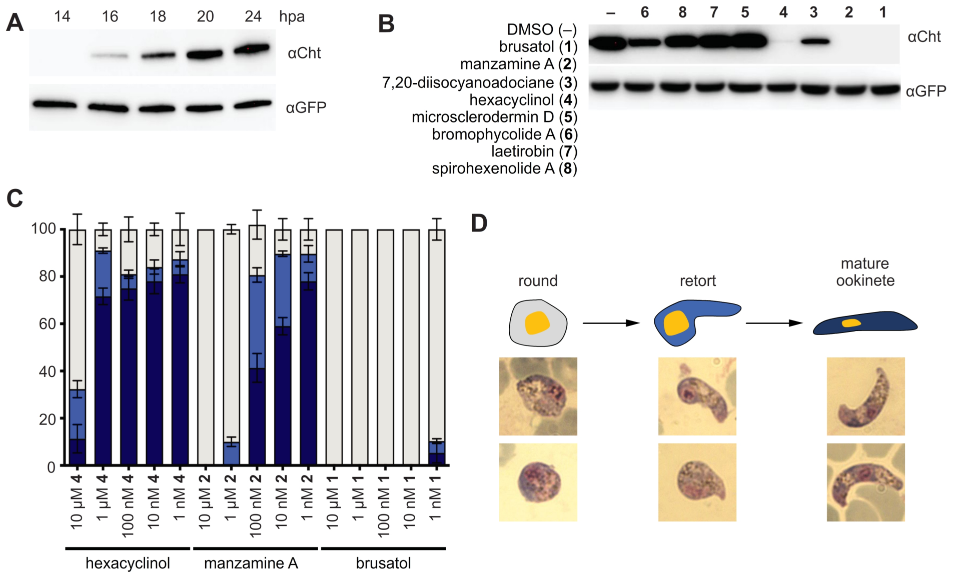

Figure S1. Effect of the compounds on ookinete development. **(A)** Chitinase protein is detected from 16 h post-activation of gametocytes and expression stabilizes by 20 h. Reporter GFP expression is maintained through development. (**B**) Western blot detecting the expression of the protease chitinase upon compound treatment. **(C-D)** Specific stages of ookinete development were assessed on exposure to manzamine A, hexacyclinol and brusatol. After fertilization of male and female gametes to form a round zygote, a complex developmental program is set in motion where the zygote undergoes meiosis, followed by establishment on an apical complex which develops into a retort and finally over 22-24 h forms a motile banana shaped ookinete capable of infecting the mosquito midgut. Mature gametocytes were exposed to varying concentrations of compounds for 30 min at 37°C followed by addition of media to mimic mosquito conditions (ookinete media). Twenty-four hours later ookinete developed was assessed by Giemsa staining followed by light microscopy. Representative images of phenotypes scored are shown.

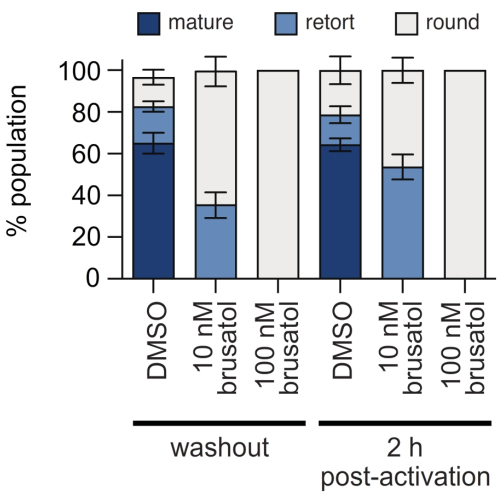

Figure S2. Multistage activity of brusatol (**1**). Ookinete development was assessed on exposure to brusatol at distinct stages (i) after a 30 min incubation with gametocytes, brusatol was removed during activation (washout) and (ii) brusatol was added 2 h post-activation.

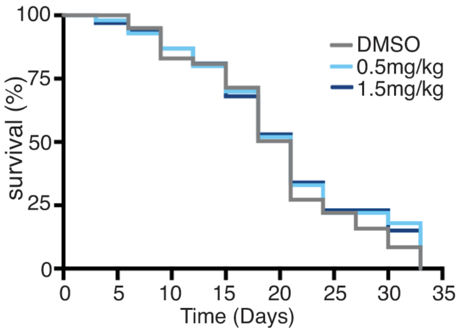

Figure S3. Mosquito survival on exposure to brusatol. Survival rate of mosquitoes fed on *P. berghei* infected mice treated with brusatol or DMSO until 33 days post-exposure (N = 100 for each condition). Mortality rate was monitored every three days. Survival analysis was performed on GraphPad prism and curves compared by Log-rank (Mantel-Cox) test. P value = 0.4659.
